# Supplementary material for: Hot spot prediction in protein-protein interactions by an ensemble system
Source: BMC Syst Biol. 2018 Dec 31;12(Suppl 9):132. doi: 10.1186/s12918-018-0665-8 (PMC6311905; doi:10.1186/s12918-018-0665-8)
Supplement: Supplementary file 2 — All relevant MATLAB code. A simple Matlab implement of our predictor. (ZIP 19,742 kb) [file 12918_2018_665_MOESM2_ESM.zip › Additional file 2/Read ASA/ASA Property/SKEMPI/1EAWA.txt]

 NetSurfP - Protein Surface Accessibility and Secondary Structure Predictions

  

|  |  |  |
| --- | --- | --- |
|  |  | NetSurfP - Protein Surface Accessibility and Secondary Structure Predictions  Technical University of Denmark |

  


---

```
# For publication of results, please cite:
# A generic method for assignment of reliability scores applied to solvent accessibility predictions.
# Bent Petersen, Thomas Nordahl Petersen, Pernille Andersen, Morten Nielsen and Claus Lundegaard
# BMC Structural Biology 2009, 9:51 doi:10.1186/1472-6807-9-51
#
# Column 1: Class assignment - B for buried or E for Exposed - Threshold: 25% exposure, but not based on RSA
# Column 2: Amino acid
# Column 3: Sequence name
# Column 4: Amino acid number
# Column 5: Relative Surface Accessibility - RSA
# Column 6: Absolute Surface Accessibility
# Column 7: Z-fit score for RSA prediction
# Column 8: Probability for Alpha-Helix
# Column 9: Probability for Beta-strand
# Column 10: Probability for Coil
E V  1EAWA                  1    0.313  48.077  -2.296   0.005   0.045   0.951
B V  1EAWA                  2    0.153  23.485  -2.573   0.004   0.514   0.481
E G  1EAWA                  3    0.376  29.591  -1.051   0.005   0.336   0.660
B G  1EAWA                  4    0.267  20.981  -1.451   0.020   0.205   0.775
E T  1EAWA                  5    0.550  76.216   1.123   0.020   0.205   0.775
E D  1EAWA                  6    0.591  85.163   1.215   0.020   0.205   0.775
B A  1EAWA                  7    0.056   6.171  -0.148   0.018   0.088   0.893
E D  1EAWA                  8    0.538  77.511   1.291   0.018   0.047   0.935
E E  1EAWA                  9    0.500  87.385   0.125   0.018   0.047   0.935
E G  1EAWA                 10    0.514  40.420  -0.434   0.018   0.019   0.964
E E  1EAWA                 11    0.484  84.537   0.943   0.018   0.047   0.935
B W  1EAWA                 12    0.157  37.710   1.194   0.019   0.141   0.840
B P  1EAWA                 13    0.106  15.027   0.883   0.005   0.336   0.660
B W  1EAWA                 14    0.056  13.420   0.704   0.018   0.846   0.136
B Q  1EAWA                 15    0.054   9.573   0.635   0.011   0.918   0.071
B V  1EAWA                 16    0.019   2.966   1.035   0.006   0.962   0.032
B S  1EAWA                 17    0.028   3.305   0.269   0.006   0.962   0.032
B L  1EAWA                 18    0.017   3.149   1.367   0.001   0.959   0.040
B H  1EAWA                 19    0.105  19.136   0.815   0.001   0.959   0.040
B A  1EAWA                 20    0.170  18.712   0.151   0.002   0.816   0.182
E L  1EAWA                 21    0.433  79.209   0.094   0.005   0.262   0.733
E G  1EAWA                 22    0.705  55.452  -2.428   0.005   0.015   0.979
E Q  1EAWA                 23    0.573 102.320  -1.321   0.005   0.015   0.979
E G  1EAWA                 24    0.367  28.899   0.409   0.004   0.138   0.858
B H  1EAWA                 25    0.101  18.354  -0.689   0.004   0.420   0.576
B I  1EAWA                 26    0.104  19.185   0.413   0.003   0.718   0.279
B C  1EAWA                 27    0.014   1.938   1.114   0.003   0.718   0.279
B G  1EAWA                 28    0.043   3.345  -1.069   0.023   0.655   0.322
B A  1EAWA                 29    0.033   3.626  -0.939   0.021   0.756   0.223
B S  1EAWA                 30    0.051   5.989   0.164   0.018   0.846   0.136
B L  1EAWA                 31    0.050   9.063   0.223   0.021   0.756   0.223
B I  1EAWA                 32    0.059  10.841   0.466   0.021   0.451   0.528
B S  1EAWA                 33    0.140  16.361   0.842   0.019   0.141   0.840
E P  1EAWA                 34    0.629  89.255   0.502   0.018   0.019   0.964
E N  1EAWA                 35    0.317  46.453   0.917   0.018   0.088   0.893
B W  1EAWA                 36    0.065  15.705   1.358   0.023   0.655   0.322
B L  1EAWA                 37    0.022   3.992   1.236   0.011   0.918   0.071
B V  1EAWA                 38    0.024   3.643   1.539   0.011   0.918   0.071
B S  1EAWA                 39    0.030   3.504   0.552   0.011   0.918   0.071
B A  1EAWA                 40    0.022   2.380   0.796   0.018   0.846   0.136
B A  1EAWA                 41    0.028   3.119  -0.317   0.023   0.655   0.322
B H  1EAWA                 42    0.059  10.714   0.652   0.023   0.655   0.322
B C  1EAWA                 43    0.014   1.966   1.544   0.079   0.592   0.329
B Y  1EAWA                 44    0.045   9.659   0.606   0.069   0.386   0.545
B I  1EAWA                 45    0.250  46.287   0.441   0.064   0.216   0.721
E D  1EAWA                 46    0.334  48.086  -0.042   0.052   0.084   0.864
E D  1EAWA                 47    0.407  58.692  -0.599   0.053   0.043   0.903
E R  1EAWA                 48    0.508 116.309  -0.831   0.053   0.043   0.903
E G  1EAWA                 49    0.510  40.106  -0.947   0.052   0.084   0.864
E F  1EAWA                 50    0.473  95.031  -1.332   0.053   0.043   0.903
E R  1EAWA                 51    0.517 118.347  -1.222   0.018   0.088   0.893
E Y  1EAWA                 52    0.368  78.727  -1.319   0.018   0.088   0.893
E S  1EAWA                 53    0.380  44.548  -1.132   0.018   0.088   0.893
E D  1EAWA                 54    0.446  64.297   0.031   0.005   0.045   0.951
B P  1EAWA                 55    0.185  26.209  -1.365   0.004   0.085   0.910
E T  1EAWA                 56    0.374  51.846  -0.701   0.005   0.262   0.733
E Q  1EAWA                 57    0.281  50.097   1.071   0.003   0.718   0.279
B W  1EAWA                 58    0.032   7.648   0.553   0.001   0.900   0.099
B T  1EAWA                 59    0.149  20.639   1.878   0.001   0.959   0.040
B A  1EAWA                 60    0.015   1.675   1.483   0.001   0.959   0.040
B F  1EAWA                 61    0.082  16.518   1.275   0.001   0.959   0.040
B L  1EAWA                 62    0.024   4.358   0.790   0.001   0.959   0.040
B G  1EAWA                 63    0.046   3.604  -0.526   0.002   0.816   0.182
B L  1EAWA                 64    0.186  34.057  -0.180   0.023   0.655   0.322
B H  1EAWA                 65    0.150  27.230   0.616   0.023   0.655   0.322
E D  1EAWA                 66    0.289  41.630  -0.160   0.022   0.552   0.426
B Q  1EAWA                 67    0.154  27.433  -0.169   0.022   0.359   0.619
E S  1EAWA                 68    0.418  48.943  -0.983   0.021   0.279   0.699
E Q  1EAWA                 69    0.489  87.335   0.117   0.020   0.205   0.775
E R  1EAWA                 70    0.506 115.851  -0.822   0.019   0.141   0.840
E S  1EAWA                 71    0.652  76.368  -1.429   0.018   0.088   0.893
E A  1EAWA                 72    0.580  63.971  -1.935   0.018   0.047   0.935
E P  1EAWA                 73    0.554  78.627  -0.655   0.018   0.047   0.935
E G  1EAWA                 74    0.515  40.530  -0.491   0.004   0.197   0.799
E V  1EAWA                 75    0.327  50.337  -0.779   0.004   0.616   0.381
E Q  1EAWA                 76    0.243  43.382   0.570   0.001   0.900   0.099
E E  1EAWA                 77    0.444  77.584   0.638   0.001   0.959   0.040
B R  1EAWA                 78    0.102  23.358   1.826   0.001   0.959   0.040
E R  1EAWA                 79    0.397  90.867   1.356   0.001   0.900   0.099
B L  1EAWA                 80    0.032   5.932  -0.153   0.002   0.816   0.182
E K  1EAWA                 81    0.427  87.834   1.119   0.001   0.900   0.099
E R  1EAWA                 82    0.305  69.937   1.240   0.001   0.959   0.040
B I  1EAWA                 83    0.057  10.453   0.535   0.001   0.959   0.040
B I  1EAWA                 84    0.126  23.292   0.909   0.001   0.959   0.040
B S  1EAWA                 85    0.189  22.151   0.521   0.002   0.816   0.182
B H  1EAWA                 86    0.066  11.951   0.798   0.004   0.197   0.799
E P  1EAWA                 87    0.490  69.517   0.204   0.005   0.015   0.979
E F  1EAWA                 88    0.497  99.728  -0.203   0.005   0.015   0.979
B F  1EAWA                 89    0.098  19.729  -0.242   0.005   0.045   0.951
E N  1EAWA                 90    0.364  53.231   0.549   0.018   0.047   0.935
E D  1EAWA                 91    0.540  77.800  -0.820   0.018   0.019   0.964
E F  1EAWA                 92    0.664 133.285  -1.568   0.018   0.019   0.964
E T  1EAWA                 93    0.474  65.785  -0.298   0.018   0.019   0.964
B F  1EAWA                 94    0.206  41.404   0.297   0.018   0.047   0.935
E D  1EAWA                 95    0.466  67.179  -0.328   0.018   0.047   0.935
B Y  1EAWA                 96    0.096  20.472   0.594   0.018   0.088   0.893
B D  1EAWA                 97    0.040   5.764  -0.269   0.022   0.359   0.619
B I  1EAWA                 98    0.027   4.921   1.063   0.018   0.846   0.136
B A  1EAWA                 99    0.018   1.973   1.270   0.011   0.918   0.071
B L  1EAWA                100    0.019   3.497   1.490   0.011   0.918   0.071
B L  1EAWA                101    0.028   5.072   1.031   0.006   0.962   0.032
B E  1EAWA                102    0.205  35.761   1.755   0.001   0.900   0.099
B L  1EAWA                103    0.033   6.134  -0.067   0.002   0.816   0.182
E E  1EAWA                104    0.475  83.052   1.183   0.022   0.359   0.619
E K  1EAWA                105    0.500 102.871   0.748   0.021   0.279   0.699
E P  1EAWA                106    0.413  58.576   0.333   0.021   0.279   0.699
B A  1EAWA                107    0.062   6.810   0.141   0.022   0.359   0.619
E E  1EAWA                108    0.586 102.304   1.373   0.021   0.279   0.699
E Y  1EAWA                109    0.297  63.448   0.001   0.020   0.205   0.775
E S  1EAWA                110    0.343  40.200  -0.605   0.018   0.047   0.935
E S  1EAWA                111    0.690  80.903  -0.154   0.005   0.045   0.951
E M  1EAWA                112    0.334  66.813   0.595   0.005   0.262   0.733
B V  1EAWA                113    0.046   7.116   0.341   0.004   0.616   0.381
B R  1EAWA                114    0.245  56.128   1.161   0.003   0.718   0.279
B P  1EAWA                115    0.095  13.410   0.269   0.018   0.846   0.136
B I  1EAWA                116    0.025   4.532   0.928   0.018   0.846   0.136
B C  1EAWA                117    0.089  12.510  -0.367   0.003   0.718   0.279
B L  1EAWA                118    0.038   6.976   0.762   0.005   0.262   0.733
B P  1EAWA                119    0.083  11.849   0.164   0.018   0.019   0.964
E D  1EAWA                120    0.460  66.286   0.956   0.018   0.019   0.964
E A  1EAWA                121    0.493  54.307  -0.522   0.018   0.019   0.964
E S  1EAWA                122    0.724  84.806  -1.905   0.018   0.019   0.964
E H  1EAWA                123    0.323  58.736  -0.019   0.018   0.047   0.935
E V  1EAWA                124    0.580  89.146   0.280   0.019   0.141   0.840
B F  1EAWA                125    0.078  15.594   0.179   0.018   0.088   0.893
E P  1EAWA                126    0.526  74.625   0.467   0.018   0.019   0.964
E A  1EAWA                127    0.641  70.649   0.056   0.018   0.019   0.964
E G  1EAWA                128    0.557  43.844  -1.092   0.005   0.015   0.979
B K  1EAWA                129    0.241  49.656   0.286   0.005   0.045   0.951
E A  1EAWA                130    0.347  38.272   1.702   0.004   0.616   0.381
B I  1EAWA                131    0.016   2.923   1.378   0.001   0.900   0.099
B W  1EAWA                132    0.095  22.920   1.376   0.001   0.959   0.040
B V  1EAWA                133    0.017   2.644   0.989   0.001   0.959   0.040
B T  1EAWA                134    0.053   7.323   0.314   0.001   0.900   0.099
B G  1EAWA                135    0.034   2.715  -1.486   0.002   0.816   0.182
B W  1EAWA                136    0.113  27.249   0.003   0.021   0.451   0.528
B G  1EAWA                137    0.055   4.352  -1.664   0.021   0.451   0.528
E H  1EAWA                138    0.302  54.879   0.539   0.021   0.451   0.528
B T  1EAWA                139    0.176  24.356   0.073   0.021   0.279   0.699
E Q  1EAWA                140    0.451  80.549   0.392   0.019   0.141   0.840
E Y  1EAWA                141    0.678 144.974  -0.574   0.018   0.047   0.935
E G  1EAWA                142    0.703  55.310  -1.734   0.005   0.015   0.979
E G  1EAWA                143    0.398  31.315  -1.381   0.005   0.015   0.979
E T  1EAWA                144    0.642  89.045  -0.771   0.018   0.019   0.964
E G  1EAWA                145    0.330  25.979  -0.123   0.058   0.017   0.925
B A  1EAWA                146    0.148  16.288  -0.553   0.181   0.016   0.803
E L  1EAWA                147    0.567 103.763   0.289   0.354   0.048   0.598
E I  1EAWA                148    0.255  47.249   1.701   0.386   0.097   0.517
B L  1EAWA                149    0.046   8.441   0.117   0.352   0.332   0.316
B Q  1EAWA                150    0.246  43.864   0.953   0.169   0.612   0.219
E K  1EAWA                151    0.363  74.710   1.075   0.072   0.855   0.073
B G  1EAWA                152    0.030   2.392   0.439   0.011   0.918   0.071
E E  1EAWA                153    0.480  83.908   0.562   0.018   0.846   0.136
B I  1EAWA                154    0.046   8.529   0.920   0.023   0.655   0.322
E R  1EAWA                155    0.378  86.562   0.800   0.023   0.655   0.322
B V  1EAWA                156    0.166  25.483   0.191   0.023   0.655   0.322
B I  1EAWA                157    0.166  30.655   1.002   0.004   0.616   0.381
E N  1EAWA                158    0.422  61.737   1.000   0.021   0.279   0.699
E Q  1EAWA                159    0.254  45.347   0.555   0.694   0.003   0.303
E T  1EAWA                160    0.716  99.309   1.342   0.858   0.002   0.139
E T  1EAWA                161    0.409  56.701   2.249   0.923   0.002   0.076
B C  1EAWA                162    0.015   2.050   1.179   0.879   0.010   0.111
E E  1EAWA                163    0.362  63.311   1.247   0.879   0.010   0.111
E N  1EAWA                164    0.668  97.854   1.107   0.879   0.010   0.111
E L  1EAWA                165    0.298  54.472   1.037   0.858   0.002   0.139
B L  1EAWA                166    0.130  23.785   0.299   0.694   0.003   0.303
E P  1EAWA                167    0.626  88.801   0.273   0.321   0.003   0.675
E Q  1EAWA                168    0.626 111.714  -0.659   0.246   0.004   0.750
E Q  1EAWA                169    0.457  81.692   1.447   0.184   0.043   0.773
B I  1EAWA                170    0.053   9.731   0.166   0.113   0.043   0.844
E T  1EAWA                171    0.266  36.936   1.197   0.018   0.019   0.964
E P  1EAWA                172    0.660  93.597   0.504   0.018   0.019   0.964
E R  1EAWA                173    0.346  79.188   0.309   0.018   0.019   0.964
B M  1EAWA                174    0.038   7.544  -0.046   0.021   0.279   0.699
B M  1EAWA                175    0.037   7.304   0.381   0.022   0.552   0.426
B C  1EAWA                176    0.016   2.303   0.893   0.023   0.655   0.322
B V  1EAWA                177    0.027   4.227   0.089   0.021   0.451   0.528
B G  1EAWA                178    0.097   7.673  -0.514   0.020   0.205   0.775
B F  1EAWA                179    0.222  44.636   0.507   0.019   0.141   0.840
E L  1EAWA                180    0.589 107.901   0.118   0.018   0.047   0.935
E S  1EAWA                181    0.752  88.134  -0.007   0.018   0.019   0.964
E G  1EAWA                182    0.476  37.469  -1.332   0.018   0.019   0.964
E G  1EAWA                183    0.459  36.155  -0.973   0.018   0.047   0.935
E V  1EAWA                184    0.434  66.706   0.553   0.019   0.141   0.840
B D  1EAWA                185    0.112  16.082  -2.250   0.020   0.205   0.775
B S  1EAWA                186    0.133  15.552  -0.000   0.020   0.205   0.775
B C  1EAWA                187    0.035   4.900  -0.986   0.021   0.279   0.699
E Q  1EAWA                188    0.368  65.796   0.215   0.020   0.205   0.775
B G  1EAWA                189    0.251  19.730  -1.398   0.018   0.088   0.893
B D  1EAWA                190    0.227  32.682  -0.034   0.018   0.047   0.935
B S  1EAWA                191    0.219  25.690  -2.013   0.018   0.019   0.964
B G  1EAWA                192    0.183  14.378  -2.591   0.018   0.047   0.935
B G  1EAWA                193    0.096   7.555  -0.726   0.018   0.088   0.893
B P  1EAWA                194    0.090  12.743  -0.801   0.020   0.205   0.775
B L  1EAWA                195    0.061  11.151   0.102   0.023   0.655   0.322
B S  1EAWA                196    0.077   8.966   0.116   0.021   0.756   0.223
B S  1EAWA                197    0.040   4.747   0.162   0.021   0.756   0.223
E V  1EAWA                198    0.422  64.861   0.180   0.004   0.616   0.381
E E  1EAWA                199    0.359  62.717  -0.476   0.005   0.262   0.733
E A  1EAWA                200    0.609  67.068  -0.840   0.004   0.085   0.910
E D  1EAWA                201    0.730 105.135  -0.704   0.005   0.015   0.979
E G  1EAWA                202    0.404  31.763  -0.570   0.004   0.085   0.910
E R  1EAWA                203    0.301  68.952   1.805   0.004   0.514   0.481
B I  1EAWA                204    0.081  14.929   0.752   0.001   0.900   0.099
B F  1EAWA                205    0.110  21.977   1.708   0.001   0.959   0.040
B Q  1EAWA                206    0.025   4.501   0.579   0.001   0.959   0.040
B A  1EAWA                207    0.050   5.543   1.311   0.001   0.959   0.040
B G  1EAWA                208    0.032   2.526  -0.398   0.011   0.918   0.071
B V  1EAWA                209    0.032   4.888   0.634   0.018   0.846   0.136
B V  1EAWA                210    0.037   5.687   0.418   0.021   0.756   0.223
B S  1EAWA                211    0.044   5.157  -0.281   0.021   0.756   0.223
B W  1EAWA                212    0.145  34.897   0.019   0.021   0.451   0.528
B G  1EAWA                213    0.101   7.957  -1.135   0.064   0.216   0.721
E D  1EAWA                214    0.347  49.988   0.501   0.056   0.142   0.802
E G  1EAWA                215    0.326  25.633  -1.218   0.052   0.084   0.864
B C  1EAWA                216    0.068   9.519  -0.425   0.053   0.043   0.903
B A  1EAWA                217    0.253  27.848  -0.842   0.058   0.017   0.925
E Q  1EAWA                218    0.467  83.460   0.689   0.018   0.019   0.964
E R  1EAWA                219    0.553 126.683   0.249   0.018   0.019   0.964
E N  1EAWA                220    0.651  95.306  -1.192   0.003   0.003   0.994
E K  1EAWA                221    0.290  59.591   0.858   0.005   0.015   0.979
B P  1EAWA                222    0.130  18.390  -1.023   0.018   0.047   0.935
B G  1EAWA                223    0.053   4.187  -1.744   0.022   0.359   0.619
B V  1EAWA                224    0.039   6.010   0.080   0.021   0.756   0.223
B Y  1EAWA                225    0.098  20.900   0.337   0.021   0.756   0.223
B T  1EAWA                226    0.039   5.479  -0.020   0.087   0.683   0.230
B R  1EAWA                227    0.221  50.655   0.651   0.074   0.484   0.442
B L  1EAWA                228    0.038   7.049   0.192   0.428   0.171   0.402
E P  1EAWA                229    0.284  40.257   0.359   0.622   0.015   0.363
E L  1EAWA                230    0.351  64.286   0.910   0.717   0.014   0.269
B F  1EAWA                231    0.067  13.407   0.018   0.802   0.014   0.185
B R  1EAWA                232    0.201  46.006   1.210   0.879   0.010   0.111
E D  1EAWA                233    0.555  80.004   1.465   0.923   0.002   0.076
B W  1EAWA                234    0.116  27.874   0.967   0.970   0.001   0.030
B I  1EAWA                235    0.037   6.808  -0.378   0.970   0.001   0.030
E K  1EAWA                236    0.416  85.468   1.275   0.970   0.001   0.030
E E  1EAWA                237    0.641 112.035   1.466   0.970   0.001   0.030
B N  1EAWA                238    0.282  41.256   1.382   0.858   0.002   0.139
B T  1EAWA                239    0.215  29.793  -0.162   0.600   0.003   0.397
E G  1EAWA                240    0.730  57.412   0.404   0.181   0.016   0.803
E V  1EAWA                241    0.553  85.027  -0.871   0.003   0.003   0.994

Explain the output.  Go back.
```

---
